# Prognostic Value of Systemic Immune-Inflammation Index in Predicting Premature Saphenous Vein Graft Disease in Patients With Coronary Artery Bypass Grafting

**DOI:** 10.7759/cureus.42833

**Published:** 2023-08-02

**Authors:** Mustafa Oguz, Akin Torun

**Affiliations:** 1 Department of Cardiology, Sultan II. Abdulhamid Han Training and Research Hospital, Istanbul, TUR

**Keywords:** chronic inflammation, coronary bypass, graft patency, saphenous vein graft disease, systemic immune-inflammation index

## Abstract

Background: Systemic inflammation is a risk factor for premature coronary artery disease (CAD), and systemic immune-inflammation index (SII), a new marker of systemic inflammation, is linked to the severity and prognosis of CAD. However, the prognosis of the SII in bypass patients' venous saphenous grafts has not been adequately evaluated. This study aimed to evaluate the prognostic value of SII in predicting premature saphenous vein graft disease (SVGD) in patients who underwent bypass surgery with venous saphenous grafts.

Methods: We retrospectively included 422 patients who had saphenous vein grafts (SVG) at least one year after bypass surgery. Of these, 222 patients had SVGD, and 200 had patent SVG.

Results: SII was higher in the SVGD group than in the control group (631.55 ± 397.84, 421.71 ± 351.07, P=0.001). A receiver operating characteristic (ROC) analysis was performed to identify the optimal cutoff point with the highest sensitivity and specificity. The optimal cutoff point for SII was defined as 430. Using a cutoff level of >430, SII predicted SVGD with a sensitivity of 73% and specificity of 56%.

Conclusion: Our study demonstrated that SII was substantially higher in patients with SVGD than in those with patent SVG. SII predicted SVGD in bypass surgery patients. SII may be a helpful parameter for identifying patients at high risk of SVGD and guiding preventive treatments.

## Introduction

Coronary artery bypass grafting (CABG) is the most performed adult cardiac surgery procedure [1]. Although arterial conduits are becoming more popular, saphenous vein grafts (SVG) are still frequently used in CABG since they are readily available, especially in emergency situations. The long-term efficacy of the CABG is an important problem such that the patency rates of SVG are 93%, 74%, and 41% after one, five, and 10 years, respectively [2]. Even before discharge from the hospital, there is a 10% risk of occlusion [3]. Although not fully understood, there are three main pathophysiological mechanisms in saphenous vein graft disease (SVGD). These are thrombosis (in the first one month), intima hyperplasia (within one month to one year after surgery), and accelerated atherosclerosis (late stage) [4]. A number of risk factors have been associated with reduced vein graft patency. Risk factors associated with SVGD include surgery-related factors, smoking, diabetes mellitus, hyperlipidemia, hypertension, chronic kidney disease, and native vessel diameter [5,6]. Inflammatory mechanisms have also been implicated in studies completed in recent years, not only in native coronary artery disease (CAD) but also in SVGD in bypass patients [7,8]. The prognosis of systemic immune-inflammation index (SII) in bypass patients' venous saphenous grafts, however, has not been adequately evaluated. This study aimed to evaluate the prognostic value of SII in predicting premature SVGD in patients who underwent bypass surgery with venous saphenous grafts.

## Materials and methods

Study subjects

The coronary angiography archive of our clinic was reviewed retrospectively between January 2017 and May 2022, and patients with SVG remained for further analysis. The patients who underwent CABG at least one year ago were included in this study. Patients with acute coronary syndrome, graft failure at one year, active cancer, decompensated heart failure, and rheumatic disease were excluded from the study. After evaluation for inclusion and exclusion criteria, 422 patients with SVG remained for final analysis. Afterward, we divided the patients into two groups according to the presence or absence of SVGD. Approval was obtained from the local Clinical Research Ethics Committee after all required documents were submitted.

Coronary angiography

The patients with stenosis greater than 50% in at least one SVG with significant stenosis were in the study group. Occlusion in the proximal and distal anastomosis lines was not considered degeneration. Patients with saphenous disease due to surgical causes were excluded from the study. Patients with patent saphenous veins were categorized as the control group.

Parameters

In this study, we evaluated patients' demographics, medical treatments, and clinical characteristics, as well as SII. SII made from the blood samples was collected 24 hours before the angiography of the patients. SII was calculated by multiplying neutrophil by platelet and dividing by lymphocyte (SII = (P × N) /L).

Statistical analysis

The statistical analysis was conducted on a dataset using SPSS Statistics (IBM Corp. Released 2012. IBM SPSS Statistics for Windows, Version 21.0. Armonk, NY: IBM Corp.). The Kolmogorov-Smirnov test was used to determine the normality of variables. If the variables were regularly distributed, means and standard deviations were used to represent them; otherwise, medians and interquartile ranges were employed. Categorical data were expressed as numbers and percentages and compared between groups using the chi-squared or Fisher's exact test. Depending on the distribution of the data, continuous variables were compared between groups using the Student's t-test or the Mann-Whitney U test. A two-way ANOVA was used to examine the impact of SII on SVGD. The significance threshold for the analysis was set at less than 0.05.

## Results

The SVGD group included 222 patients, while the control group included 200 individuals without degenerated saphenous grafts. The basal characteristics of the groups were presented in Table 1.

**Table 1:** Basal characteristics of the patients during the index coronary angiography ACE/ARB: angiotensin-converting enzyme/angiotensin receptor blocker, SVGD: saphenous vein graft disease a: patients with patent SVG without degeneration

	SVGD (N=222)	Control group^a ^(N=200)	p-value
Male gender, n (%)	192 (86.5)	175 (87.5)	0.757
Age, years (mean±SD)	66.32+9.48	67.38+10.38	0.329
Hypertension, n (%)	187 (84.2)	160 (80)	0.256
Diabetes mellitus, n (%)	121 (54.4)	91 (45.5)	0.065
Hyperlipidemia, n (%)	148 (66.7)	114 (57)	0.041
Peripheral arterial disease, n (%)	20 (9)	22 (11)	0.495
Chronic kidney disease, n (%)	65 (29.3)	40 (20)	0.028
Cerebrovascular accident, n (%)	14 (6.3)	19 (9.5)	0.222
Smoking, n (%)			
Active	70 (31.5)	48 (24)	0,312
Ex-smoker	20 (9)	12 (6)	
Never	132 (59.5)	140 (70)	
Medical treatment, n (%)			
Antiaggregan	201 (90.5)	182 (91)	0.817
Anticoagulant	25 (11.3)	17 (8.5)	0.344
Beta blocker	178 (80.2)	149 (74.5)	0.163
ACE/ARB	148 (66.7)	93 (46.5)	0,001
Spironolactone	46 (20.7)	17 (8.5)	0,001
Statin	140 (63.1)	127 (63.5)	0.423
Calcium channel blocker	48 (21.6)	35 (17.5)	0.228

The mean age was 66.3 ± 9.4 years in the SVGD group and 67.3 ± 10.3 years in patients with patent SVG (P=0.329). The results of the two groups were similar in terms of hypertension, diabetes mellitus, peripheral arterial disease, and cerebrovascular accident compared to chronic disease. Hyperlipidemia was significantly higher in the SVGD group than in the control group (66.7% vs 57.0%, p=0.041, respectively). Another significant difference between the groups was chronic kidney disease, and it was higher in the SVGD group (65 (29.3%), 40 (20%), P=0.029).

The use of antiaggregant, anticoagulant, beta-blocker, ACE/ARB, spironolactone statin, and calcium channel blockers in medical treatment was investigated. A significant difference was observed in ACE/ARB and spironolactone treatments (P=0.003, 0.001, respectively).

In terms of the age of SVG, the time elapsed after surgery was observed more in the SVGD group. While it was 8.67 ± 6.76 years in the SVGD group, it was 7.20 ± 5.90 years in the control group (P= 0.018).

**Figure 1:**
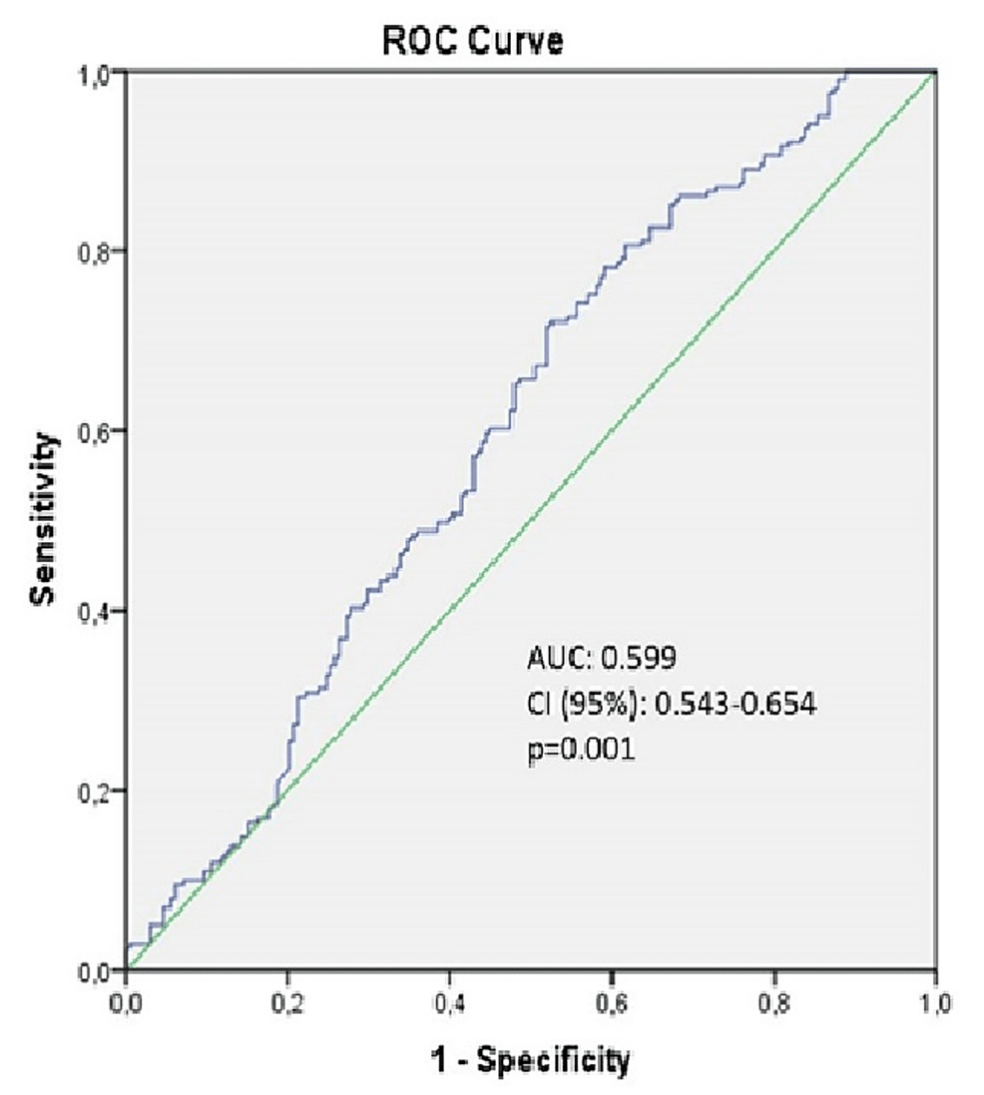
ROC curves of SII predicting SVGD ROC: receiver operating characteristic, AUC: area under the ROC curve, CI: confidence interval

Table 2 shows the graft detail and laboratory parameters for each group. In the SVDG group, the mean number of saphenous grafts was 2.09 ± 0.84, with 1.50 ± 0.697 of these saphenous grafts degenerated. The mean number of saphenous grafts in the control group was 1.49±0.82. Hemoglobin, white blood cell, platelet, total cholesterol, high-density lipoprotein (HDL), low-density lipoprotein (LDL), and triglyceride parameters did not differ between the two groups. The SII was significantly different between the two groups. SII was higher in the SVGD group than in the control group (631.55 ± 397.84, 421.71 ± 351.07, P=0.001). ROC analysis was performed to identify the optimal cutoff point with the highest sensitivity and specificity. The optimal cutoff point for SII was defined as 430. Using a cutoff level of >430, SII predicted SVGD with a sensitivity of 73% and specificity of 56% (Figure 1).

**Table 2:** Graft details and laboratory parameters HDL: high-density lipoprotein, LDL: low-density lipoprotein, SII: systemic immune-inflammation index, SVGD: saphenous vein graft disease, WBC: white blood cell a: patients with patent SVG

	SVDG	Control group^a^	p-value
Age of SVG (mean±SD)	8.67±6.76	7.20±5.90	0.018
Saphenous graft (mean±SD)	2.09±0.84	1.49±0.82	
Degenerated saphenous (mean±SD)	1.50±0.697	0	
Hemoglobin, mg/dl (mean±SD)	13.27±1.89	13.75±1.65	0.099
WBC, x 1000µL (median, IQR)	7.57 (3.01)	7.5 (2.12)	0.274
Platelet, x 1000µL (median, IQR)	207 (90)	210.50 (75.5)	0.329
Total cholesterol, mg/dl (median, IQR)	160 (53.5)	168 (51)	0.224
LDL, mg/dl (median, IQR)	90 (35.5)	100.5 (33.5)	0.205
HDL, mg/dl (mean±SD)	43.3 ± 12.58	43.62 ± 12.31	0.875
Triglyceride, mg/dl (mean±SD)	136.09 ± 68.68	149.69 ± 78.92	0.258
SII (mean±SD)	631.55 ± 397.84	421.71 ± 351.07	0.001

In univariate logistic regression analysis, diabetes mellitus, hyperlipidemia, chronic kidney disease, ACE/ARB, spironolactone, statins, active smoking, age of the SVG, and SII remained for multivariate analysis. In multivariate regression analysis, ACE/ARB, age of the SVG, and SII were shown to be independent predictors of SVGD with a p-value of 0.001, 0.042, and 0.001, respectively (Table 3).

**Table 3:** Multivariate logistic regression analysis for SVGD ACE/ARB: angiotensin-converting enzyme/angiotensin receptor blocker, SII: systemic immune-inflammation index

Possible confounding factor	Odds ratio	95% confidence interval	p-value
Diabetes mellitus	1.319	0.858-2.025	0.207
Hyperlipidemia	1.309	0.845-2.028	0.227
Chronic kidney disease	1.497	0.910-2.461	0.112
ACE/ARB	2.034	1.312-3.152	0.001
Spironolactone	1.703	0.883-3.284	0.112
Statins	1.678	1.089-2.585	0.021
Age of SVG	1.036	1.001-1.072	0.042
Active smoking	1.088	0.671-1.765	0.732
SII >430	2.074	1.337-3.219	0.001

## Discussion

In this study, we investigated 422 patients to observe the effect of the systemic inflammatory index on saphenous veins. The study's findings demonstrated that SII predicted SVGD in bypass surgery patients.

Chronic low-grade inflammation is involved in coronary atherosclerosis, presenting multiple clinical manifestations ranging from asymptomatic to stable angina, acute coronary syndrome, heart failure, and sudden cardiac death [9]. The role of inflammation in the atherosclerotic process has been well described previously. Yang et al. investigated the relationship between SII and major cardiovascular events and compared it with traditional risk factors, and they found that the predictive power of SII was better [10]. Candemir et al. showed that SII was significantly associated with the severity of CAD and high syntax score in patients with stable angina pectoris [11]. The previously proven parallelism between syntax score and SII appears to be comparable to the association observed in our study between SVGD and SII. SII is an effective inflammatory indicator that is more prognostic than other inflammatory indicators, according to Dogdus et al. [8]. These findings show that inflammatory processes have an impact on accelerated atherosclerosis. SII has also been shown to be associated with diseases such as heart failure, valve surgery, microvascular dysfunction, pulmonary embolism, cancer, depression, and rheumatologic diseases [12-14]. To avoid bias, patients with acute coronary syndrome, active cancer, decompensated heart failure, and rheumatic disease were excluded from this study.

There is a general consensus that vein graft atherosclerosis differs from arterial lesions in terms of temporal and histological changes. Vein graft atherosclerosis is more rapid, with diffuse concentric changes and a less noticeable fibrous cap, making venous plaques more vulnerable to rupture and subsequent thrombus formation [15]. For these reasons, saphenous grafts may be more susceptible to inflammatory processes compared to coronary arteries. We observed that the SII has an effective role in saphenous vein degeneration. Doğan et al. showed the relationship between the neutrophil-to-lymphocyte ratio (NLR) and saphenous vein degeneration in their study of 120 patients [16]. In another study, Lui et al. showed that the SII is an independent risk factor for the occurrence and also severity of CAD. Additionally, the prediction of severe coronary stenosis was higher than NLR, platelet lymphocyte ratio, and CRP levels for SII [17]. Our study will make a significant contribution to the literature, as it is the first study on saphenous grafts and provides data on 422 patients. These data may be useful in the development of anti-inflammatory therapies to prevent saphenous graft disease in the future.

The 10-year patency rate of vein grafts is no more than 50-60%, and atherosclerosis is considered the main cause. In our study, we found that saphenous vein age is a factor affecting degeneration and patency, as expected. The inflammatory effect in this process may explain why the degeneration is more pronounced as the saphenous vein age increases. Our study and literature showed that SII is a reliable inflammatory marker. It also has a significant role in SVGD. Our study results are considered valuable for daily clinical practice.

Limitations

Our study has some limitations. First, it was a retrospective and single-center study, which may limit the generalizability of the results. We did not have data on the dietary habits of the patients, which may affect the SII levels and the SVGD risk. Furthermore, we did not perform a serial measurement of SII after surgery, which may provide more insight into the dynamic changes of inflammation and graft patency. Therefore, further prospective and multicenter studies with larger sample sizes and longer follow-up periods are needed to confirm our findings and to evaluate the prognostic value of SII in CABG patients.

## Conclusions

Our study demonstrated that SII was substantially higher in patients with SVGD than in those with patent SVG. SII had a moderate predictive accuracy for SVGD in patients who underwent bypass surgery with venous saphenous grafts. SII may be a helpful parameter for identifying patients at high risk of SVGD and guiding preventive treatments.

## References

[1] Fitzgibbon GM, Kafka HP, Leach AJ, Keon WJ, Hooper GD, Burton JR (1996). Coronary bypass graft fate and patient outcome: angiographic follow-up of 5,065 grafts related to survival and reoperation in 1,388 patients during 25 years. J Am Coll Cardiol.

[2] Goldman S, Zadina K, Moritz T (2004). Long-term patency of saphenous vein and left internal mammary artery grafts after coronary artery bypass surgery: results from a Department of Veterans Affairs Cooperative Study. J Am Coll Cardiol.

[3] Harskamp RE, Lopes RD, Baisden CE, de Winter RJ, Alexander JH (2013). Saphenous vein graft failure after coronary artery bypass surgery: pathophysiology, management, and future directions. Ann Surg.

[4] Parang P, Arora R (2009). Coronary vein graft disease: pathogenesis and prevention. Can J Cardiol.

[5] Gao J, Liu Y, Li YM (2018). Review of risk factors, treatment, and prevention of saphenous vein graft disease after coronary artery bypass grafting. J Int Med Res.

[6] Gökay S, Ciçek D (2012). Saphenous vein graft disease: causes, prevention, and contemporary treatment strategies (Article in Turkish). Turk Kardiyol Dern Ars.

[7] de Vries MR, Quax PH (2018). Inflammation in vein graft disease. Front Cardiovasc Med.

[8] Dogdus M, Dindas F, Yenercag M (2023). The role of systemic immune inflammation index for predicting saphenous vein graft disease in patients with coronary artery bypass grafting. Angiology.

[9] Sagris M, Theofilis P, Antonopoulos AS (2021). Inflammation in coronary microvascular dysfunction. Int J Mol Sci.

[10] Yang YL, Wu CH, Hsu PF (2020). Systemic immune-inflammation index (SII) predicted clinical outcome in patients with coronary artery disease. Eur J Clin Invest.

[11] Candemir M, Kiziltunç E, Nurkoç S, Şahinarslan A (2021). Relationship between systemic immune-inflammation index (SII) and the severity of stable coronary artery disease. Angiology.

[12] Gok M, Kurtul A (2021). A novel marker for predicting severity of acute pulmonary embolism: systemic immune-inflammation index. Scand Cardiovasc J.

[13] Tosu AR, Kalyoncuoglu M, Biter Hİ (2021). Prognostic value of systemic immune-inflammation index for major adverse cardiac events and mortality in severe aortic stenosis patients after TAVI. Medicina (Kaunas).

[14] Yaşar E, Bayramoğlu A (2022). Systemic immune-inflammation index as a predictor of microvascular dysfunction in patients with cardiac syndrome x. Angiology.

[15] Hassantash SA, Bikdeli B, Kalantarian S, Sadeghian M, Afshar H (2008). Pathophysiology of aortocoronary saphenous vein bypass graft disease. Asian Cardiovasc Thorac Ann.

[16] Doğan M, Akyel A, Cimen T (2015). Relationship between neutrophil-to-lymphocyte ratio and saphenous vein graft disease in patients with coronary bypass. Clin Appl Thromb Hemost.

[17] Liu Y, Ye T, Chen L, Jin T, Sheng Y, Wu G, Zong G (2021). Systemic immune-inflammation index predicts the severity of coronary stenosis in patients with coronary heart disease. Coron Artery Dis.

